# Increased Lymph Node Yield in Colorectal Cancer Is Not Necessarily Associated with a Greater Number of Lymph Node Positive Cancers

**DOI:** 10.1371/journal.pone.0104991

**Published:** 2014-08-13

**Authors:** Aisling O'Shea, Omar Aly, Craig N. Parnaby, Malcolm A. Loudon, Leslie M. Samuel, Graeme I. Murray

**Affiliations:** 1 Department of Pathology, Aberdeen Royal Infirmary, NHS Grampian, Aberdeen, United Kingdom; 2 Department of Pathology, Division of Applied Medicine, School of Medicine and Dentistry, University of Aberdeen, Aberdeen, United Kingdom; 3 Department of Colorectal Surgery, Aberdeen Royal Infirmary, NHS Grampian, Aberdeen, United Kingdom; 4 Department of Clinical Oncology, Aberdeen Royal Infirmary, NHS Grampian, Aberdeen, United Kingdom; University of Torino, Italy

## Abstract

The presence of lymph node metastasis is a key prognostic factor in colorectal cancer and lymph node yield is an important parameter in assessing the quality of histopathology reporting of colorectal cancer excision specimens. This study assesses the trend in lymph node evaluation over time in a single institution and the relationship with the identification of lymph node positive tumours. It compares the lymph node yield of a contemporary dataset compiled from the histopathology reports of 2178 patients who underwent surgery for primary colorectal cancer between 2005 and 2012 with that of a historic dataset compiled from the histopathology reports of 1038 patients who underwent surgery for colorectal cancer at 5 yearly intervals from 1975 to 2000. The mean lymph node yield was 14.91 in 2005 rising to 21.38 in 2012. In 2012 92.9% of all cases had at least 12 lymph nodes examined. Comparison of the mean lymph node yield and proportion of Dukes C cases shows a significant increase (Pearson correlation = 0.927, p = 0.001) in lymph node yield while there is no corresponding significant trend in the proportion of Dukes C cases (Pearson correlation = −0.138, p = 0.745). This study shows that there is increasing yield of lymph nodes from colorectal cancer excision specimens. However, this is not necessarily associated with an increase number of lymph node positive cancers. Further risk stratifying of colorectal cancer requires consideration of other pathological parameters especially the presence of extramural venous invasion and relevant biomarkers.

## Introduction

Detection of metastasis in tumour-associated lymph nodes has a highly significant effect on prognosis in patients with colorectal cancer. All patients with one or more lymph nodes displaying metastasis are upstaged to Dukes C/stage 3 disease. This has important therapeutic consequences for a patient, as all these patients are considered for treatment with adjuvant chemotherapy. [1] Clinical trials including QUASAR, MOSAIC20 and NSABP C-0721 trials have shown that adjuvant chemotherapy with 5-fluorouracil alone enhances 5-year survival by around 5–7% while a combination of 5-fluorouracil and oxaliplatin increases 5-year survival by 10–15% in stage 3 disease. [1]–[4] Consequently according to current guidelines inadequate detection of positive lymph nodes can lead to under treatment of lymph node positive colorectal cancer.

It is generally considered and accepted that examining more lymph nodes in a colorectal cancer excision specimen increases the likelihood of identifying involved lymph nodes and thus upstaging the cancer. However, recent studies have provided some contrary evidence. Data collected from the National Cancer Institute's Surveillance, Epidemiology, and End Results (SEER) database for 86,394 patients showed that while the number of lymph nodes being evaluated increased over time but that this did not significantly increase the detection of node positive cancers. [5] The study showed 34.5% of patients had 12 or more lymph nodes evaluated between 1988–1990, compared to 73.6% of patients between 2006 and 2008. However, the number of lymph node positive cancers (Dukes C/stage 3) cancers detected did not significantly increase between these time periods (40% node positive cancers in 1988–1990 vs. 42% in 2006–2008 p = 0.53). However, information was not consistently available in the SEER database regarding the use of neoadjuvant therapy or participation in a screening programme both factors which could influence lymph node yield and tumour stage. Results from other centers are similar and it has been suggested that suggest that examining more than 12–17 lymph nodes may have a marginal effect on colorectal cancer staging. [6]

These recent studies appear to contradict the hypothesis that examining more lymph nodes increases the detection rate of node positive disease. Nevertheless the studies by Parsons et al., [5], Bui et al., [7] and Chang et al., [8] all show improved survival in patients when a higher number of lymph nodes are examined compared to patients with fewer lymph nodes examined. This is even seen for lymph node negative cancers. [5], [8] These findings suggest that factors other than upstaging may account for the relationship between lymph node yield and survival. A simple explanation is the tumor-host interaction. Patients who mount a stronger immune response to their cancers may have larger numbers of lymph nodes present and of greater size, making them easier to find by pathologists. In addition to this, more extensive surgical resection of the tumour's lymphatic drainage system and thus more complete node clearance may itself result in lower rates of local or distant cancer recurrence. [9]

Despite the variation in the published evidence, guidance and standards on lymph node yield have been introduced as a quality performance indicator for the histopathology reporting of colorectal cancer resections. Professional and regulatory bodies including The Royal College of Pathologists UK, Healthcare Improvement Scotland and American College of Pathologists have recommended the identification of a minimum number of lymph nodes from colorectal cancer resections as a standard and also as a quality performance indicator. [10]–[12] The Royal College of Pathologists UK recommends a minimum of 12 lymph nodes and Healthcare Improvement Scotland guidance is for 80% of colorectal cancer resections to have 12 or more lymph node retrieved. [10], [11]

This study assesses the trend in lymph node evaluation in a large cohort of over a thirty seven year period in a single centre and focuses on the trend in lymph node yield in comparison with the proportion of lymph node positive cases.

## Methods

### Study population

This study included 3216 patients who had their colorectal cancer pathology reported by the Department of Pathology, Aberdeen Royal Infirmary from 1975–2012. The Department of Pathology at Aberdeen Royal Infirmary is the regional pathology centre serving three health authorities and four acute hospitals namely NHS Grampian (Aberdeen Royal Infirmary; an academic teaching hospital, Dr Grays' Hospital, Elgin; a district general hospital), NHS Orkney (Balfour Hospital, Kirkwall; remote and rural hospital) and NHS Shetland (Gilbert Bain Hospital, Lerwick; remote and rural hospital). Approximately 75% of the total volume of colorectal cancer surgery is performed at Aberdeen Royal Infirmary. Relevant pathological data was extracted from the pathology reports of the resected colorectal cancer excision specimens and two databases were constructed.

**Figure 1:**
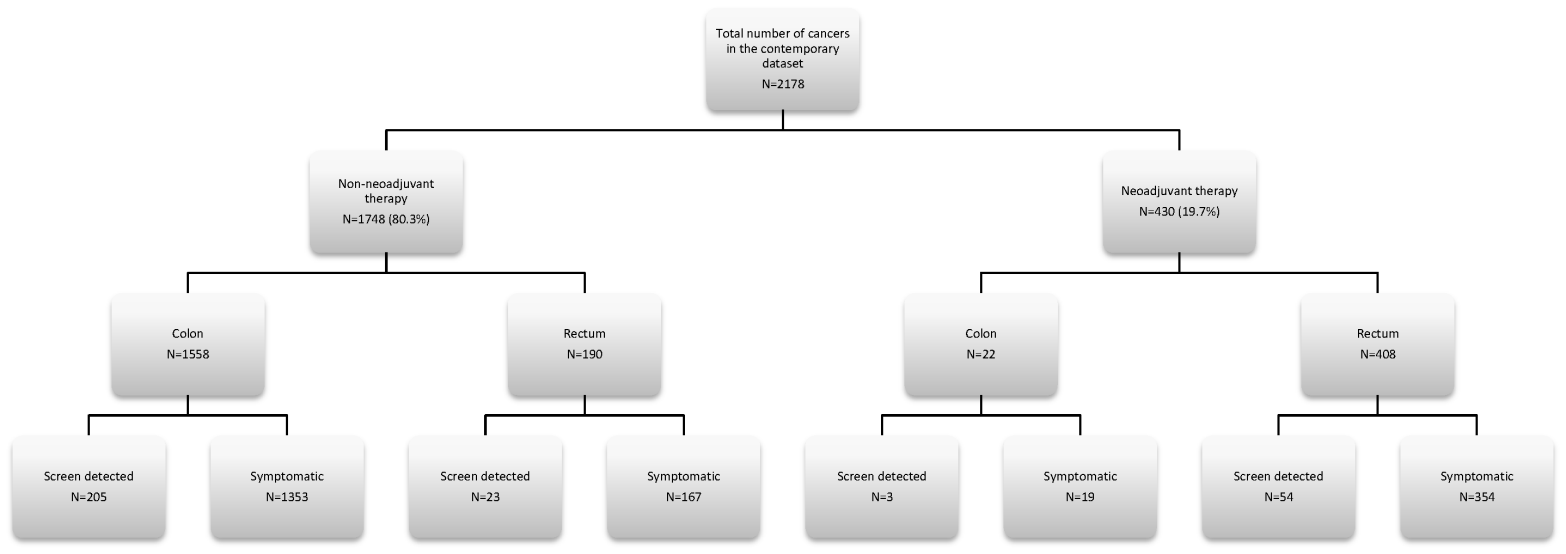
Overview of the distribution of colorectal cancers in the contemporary dataset.

The first database was compiled using prospectively collected histopathological data from colorectal cancers resected between 2005 and 2012. The information recorded in this database includes age, gender, year of operation, administration of neoadjuvant therapy, whether the tumour was screen detected, tumour site, tumour differentiation, tumour (T) stage, extramural venous invasion (EMVI), total number of lymph nodes examined, number of lymph nodes involved by metastatic tumour, nodal (N) stage and Dukes stage. Information for each parameter was available for every patient. This database is referred to as the contemporary dataset and the distribution of cases in this series is shown in figure 1. The histopathology of all the cases in this database were reported according to the criteria of the Royal College of Pathologists UK [10] for the reporting of colorectal cancer excision specimens and all these cases were subject to multi-disciplinary review. Throughout this period of time NHS Grampian has been a centre for the NHS Scotland bowel screening programme (2000–2006, pilot centre for evaluation of programme, 2007-part of national programme following its implementation throughout Scotland).

**Figure 2:**
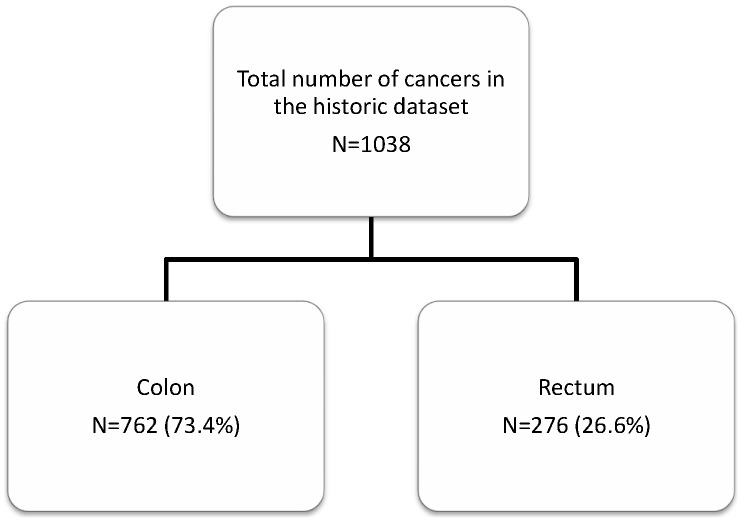
Overview of the distribution of colorectal cancers in the historic dataset.

The second database was compiled retrospectively from cases of colorectal cancers identified by searching the Department of Pathology computer database using the search terms ‘colon’, ‘rectum’ and ‘carcinoma’. Histopathology reports of colorectal cancers excised at 5 yearly intervals 1975, 1980, 1985, 1990, 1995 and 2000 were identified and reviewed. Information on patient age and gender, tumour site, tumour differentiation, T stage, N stage, total number of lymph nodes examined, total number of lymph nodes involved and Dukes stage were extracted from these reports (when stated) and used to assemble the database. When not explicitly stated Dukes stage was inferred from the information stated in the histopathology report regarding the depth of tumour invasion and lymph node involvement. This database is referred to as the historic dataset and the distribution of cases is shown in figure 2.

### Statistics

Both databases were compiled in Excel 2007 and then imported into IBM SPSS version 21 for Windows 7™ (IBM, Portsmouth, UK) to perform data analysis. Linear trends for changes in lymph node yield and proportion of Dukes C cases with time were determined using Excel 2007 (Microsoft, Seattle, USA).

### Ethics

The project was carried out using anonymised data and within the remit of ethics approval (ref. nos. 08/S0801/81) from the North of Scotland research ethics committee.

## Results

The clinico-pathological parameters collected for the contemporary and historic datasets are summarised in table 1. Information for all parameters was complete for the contemporary database where as significant proportion of pathology reports in the historic dataset had no information documented on gender (12.8%), age (10.4%), number of lymph nodes retrieved (66%) and number of lymph nodes involved by metastatic tumour (66.6%). Pathology reports in the historic dataset often made descriptive comments about the extent of tumour invasion from which tumour stage could be inferred and similarly about lymph node involvement from which Dukes stage could be inferred.

**Table 1:** Clinico-pathological parameters of the contemporary and historic colorectal cancer datasets.

		Contemporary dataset (n = 2178)			Historic dataset (n = 1038)
		All cases (n = 2178)	Non-neodjuvant therapy (n = 1748)	Neoadjuvant therapy (n = 430)	
Gender					
	Male	54.9% (1195)	53.3% (931)	61.4% (264)	42.9% (445)
	Female	45% (980)	46.6% (814)	38.6% 166	44.4.% (461)
	Unknown	0.1% (3)	0.2% (3)	-	12.8% (132)
Age					
	<71	51.2% (1114)	47.8% (836)	64.7% (278)	45.4% (471)
	≥71	48.7% (1061)	52% (909)	35.3% (152)	44.2% (459)
	Unknown	0.1% (3)	0.2% (3)	-	10.4% (108)
Tumour site					
	Proximal	41.1% (896)	50.6% (885)	2.6% (11)	43.3% (449)
	Distal	31.4% (684)	38.5% (673)	2.6% (11)	30.1% (312)
	Rectum	27.5% (598)	10.9% (190)	94.9% (408)	26.7% (277)
Tumour stage					
	T1	4.4% (96)	5.5% (96)	-	2.4% (25)
	T2	8.1% (176)	10.1% (176)	-	9.5% (99)
	T3	48.4% (1054)	60.3% (1054)	-	80.2% (832)
	T4	19.4% (422)	24.1% (422)	-	7.9% (82)
	yT0	4.5% (98)	-	22.8% (98)	-
	yT1	2% (44)	-	10.2% (44)	-
	yT2	4.2% (91)	-	21.2% (91)	-
	yT3	8.1% (177)	-	41.2% (177)	-
	yT4	0.9% (20)	-	4.7% (20)	-
Nodal stage					
	N0	43.9% (957)	54.7% (957)	-	56.1% (582)
	N1	22.5% (490)	28% (490)	-	26.9% (279)
	N2	13.8% (301)	17.2% (301)	-	11.7% (121)
	yN0	14.9% (325)	-	75.6% (325)	-
	yN1	3.4% (74)	-	17.2% (74)	-
	yN2	1.4% (31)	-	7.2% (31)	-
	Nx	-			5.4% (56)
Dukes stage					
	A	15.6% (339)	12.6% (221)	27.4% (118)	9.5% (99)
	B	39.2% (853)	42.1% (736)	27.2% (117)	46.5% (483)
	C	41.1% (895)	45.3 (791)	24.2% (104)	38.5% (400)
	Not assessed^1^	-	-	-	5.4% (56)
	Not determined^2^	4.1% (91)	-	21.2% (91)	-
Lymph node yield					
	<12nodes	18% (390)	17.4% (304)	20% (86)	28.3% (294)
	≥12 nodes	82% (1786)	82.6% (1444)	80% (344)	5.7% (59)
	Not stated	-			66% (685)

1. No lymph nodes assessed or no comment about lymph node positivity

2. Complete pathologic response (i.e. ypT0N0) to pre-operative neoadjuvant therapy.

The lymph node yield in the contemporary series of colorectal cancers is summarised in table 2 and in detail in table 3. Mean lymph node yield increased from 14.91 in 2005 to 21.38 in 2012 and the proportion of cases with 12 or more lymph nodes increased from 69.4% of cases in 2005 to 92.9% of cases in 2012. Mean lymph node yield is significantly less in the group that had received neoadjuvant therapy prior to surgery compared to those patient who did not receive any such therapy (p<0.001, Mann Whitney U test). Mean lymph node yield is also less in distal colon cancers compared with proximal colon cancers (p<0.001, Mann Whitney U test).

**Table 2:** Lymph node yield for all colorectal cancers 2005–2012.

		Mean lymph node yield	Percentage (number) of cases with ≥12 lymph nodes
All colorectal cancer cases (n = 2178)		17.4	82% (1786)
	Cases which did not receive neo-adjuvant therapy (n = 1748)	17.66	82.6% (1442)
	Cases which received neoadjuvant therapy (n = 430)	16.34	80% (344)
Colon cancer (n = 1580)		17.46	81.8% (1292)
	Proximal (n = 896)	18.28	85.7% (769)
	Distal (n = 684)	16.27	76.6% (523)
Rectal cancers (n = 598)		17.24	82.8% (495)
	Cases which did not receive neoadjuvant therapy (n = 190)	19.24	90% (171)
	Cases which received neoadjuvant therapy (n = 408)	16.32	79.4% (324)

**Table 3:** Lymph node yield in colorectal cancer analysed by year.

Year	Total number of colorectal cancers	Mean lymph node yield for all cases	Percentage (number) of all cases with ≥12 lymph nodes	Mean lymph node yield of non neoadjuvant cases	Percentage (number) of non-neoadjuvant cases with ≥12 lymph nodes	Mean lymph node yield colon cancer	Percentage (number) of colon cancer cases with ≥12 lymph nodes	Mean lymph node yield of neoadjuvant cases	Percentage (number) of neoadjuvant cases with ≥12 lymph nodes	Mean lymph node yield of bowel screening detected cases	Percentage (number) of bowel screening detected cases ≥12 LNs
1975	119	-	-	-	-			-	-	-	-
1980	179	-	-	-	-			-	-	-	-
1985	163	-	-	-	-			-	-	-	-
1990	188	-	-	-	-			-	-	-	-
1995	173	5.41	3.5% (6)	-	-			-	-	-	-
2000	216	9	24.5% (53)	-	-			-	-	-	-
2005	245	14.91	69.4% (170)	15.36 (n = 212)	71.2% (151)	15.09 (n = 182)	68.9% (125)	12.03 (n = 33)	57.6% (19)	13.62 (n = 13)	53.8% (7)
2006	244	14.94	68.9% (168)	15.09 (n = 197)	80.4%(135)	15.15 (n = 186)	70.3% (129)	14.33 (n = 47)	69.6% (33)	16.62 (n = 13)	69.2% (9)
2007	262	15.73	77.1% (202)	15.97 (n = 203)	80.8% (164)	15.89 (n = 197)	83.2% (164)	14.9 (n = 59)	64.4% (38)	14.8 (n = 5)	60% (3)
2008	298	17.22	84.2% (251)	17.62 (n = 236)	85.2% (201)	17.42 (n = 202)	84.2% (171)	15.71 (n = 62)	80.6% (50)	18.65 (n = 46)	95.6% (44)
2009	278	17.4	85.3% (237)	17.41 (n = 218)	85.3% (186)	17.32 (n = 192)	83.3% (160)	17.23 (n = 60)	86.4%(51)	16.62 (n = 37)	83.8% (31)
2010	270	16.81	83.3% (225)	16.84 (n = 205)	81.5% (167)	16.51 (n = 196)	80.6% (158)	16.71 (n = 65)	89.2% (58)	16.71 (n = 56)	82.1% (46)
2011	284	19.77	90.8% (258)	19.75 (n = 231)	89.6% (207)	19.21 (n = 206)	88.3% (182)	19.83 (n = 53)	96.2% (51)	19.30 (n = 46)	93.5% (43)
2012	297	21.38	92.9% (276)	22.02 (n = 246)	94.3% (232)	22.12 (n = 219)	94.5% (207)	18.29 (n = 51)	86.3% (44)	18.93 (n = 69)	89.9% (62)

**Figure 3:**
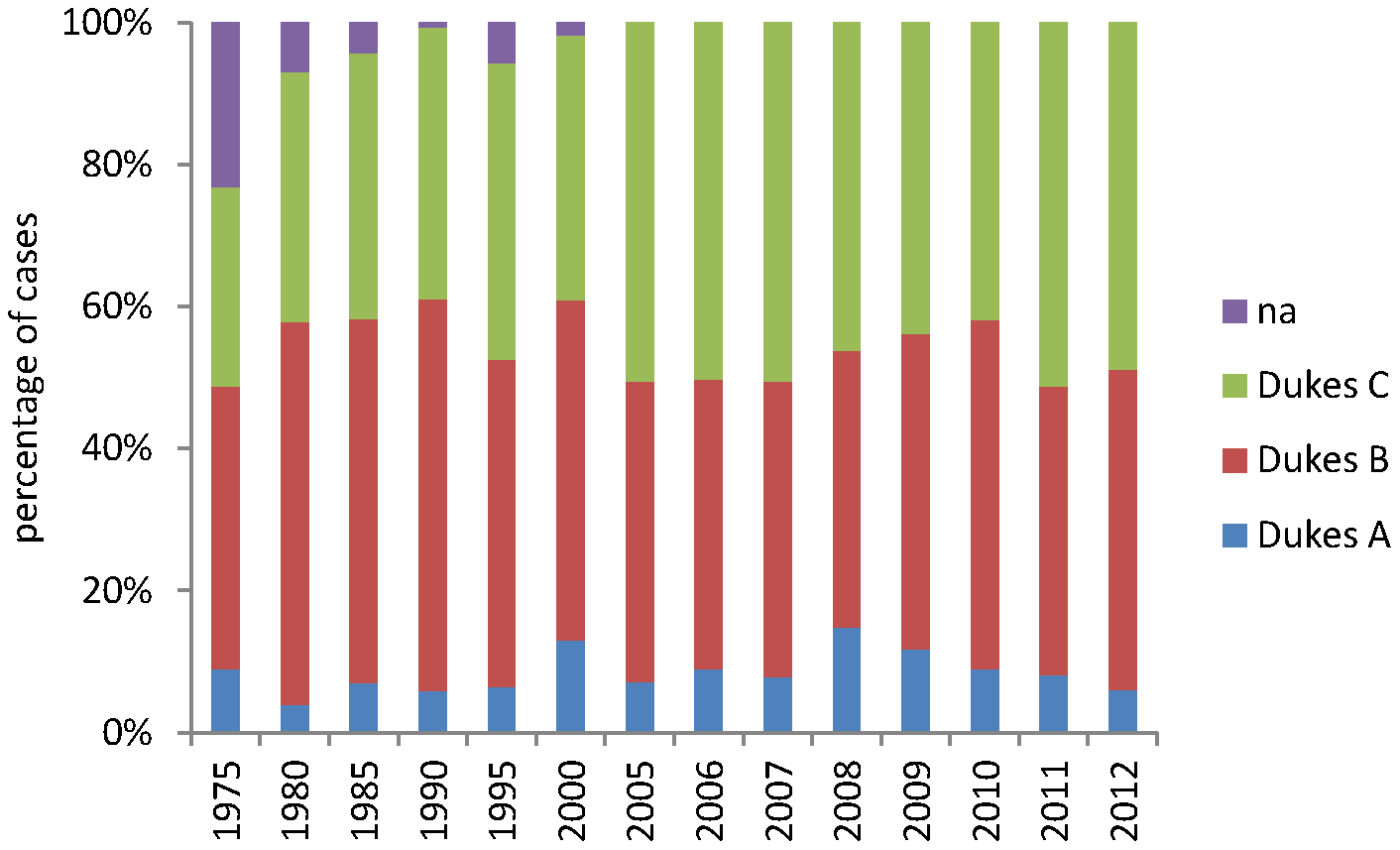
The frequency of individual tumour stages in colon cancer from 1975–2012. The 2005–2012 data excludes screen detected cancers thus allowing direct comparison with the historic data set prior to the introduction of the bowel cancer screening programme. Rectal cancers have also been excluded as the introduction of neo-adjuvant therapy for rectal cancers excludes the direct comparability of rectal cancer staging.

**Figure 4:**
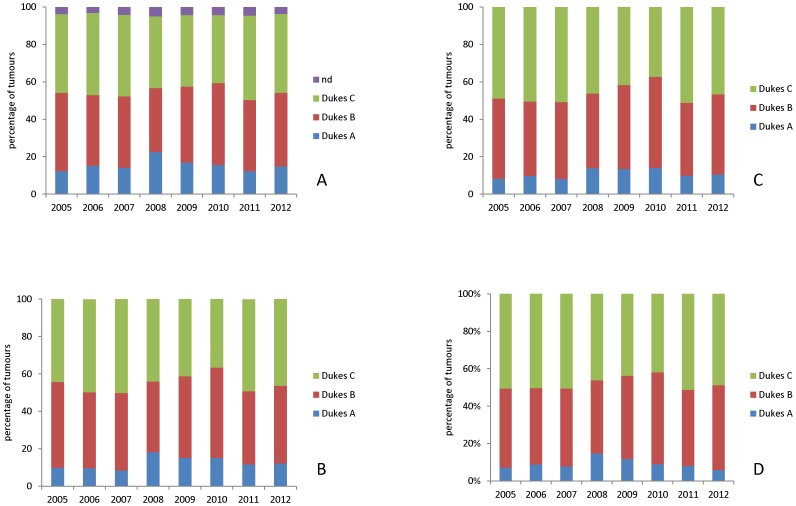
The tumour stage frequency distribution of colorectal cancer 2005–2012. A. All colorectal cancers, B. cases that did not receive neoadjuvant therapy, C. colon cancers, D. colon cancers excluding bowel screening detected cases.

The frequency distribution of tumour stage in both the historic dataset and the contemporary dataset is shown in figure 3 and in detail in table 4. Data is shown only for colon cancers with both screen detected cases and rectal cancers excluded to allow direct comparison of the historic cases with the contemporary cases. The mean frequency of Dukes C cases in the historic dataset is 36.53% of cases while the mean frequency of Dukes C cases in the contemporary dataset is 47.9% of cases. The frequency distribution of Dukes stages per year in the contemporary dataset is shown in figure 4 and illustrates for all cases (figure 4A), cases which did not receive neoadjuvant therapy (figure 4B), colon cancers (figure 4C) and symptomatic colon cancers (i.e. bowel cancer screening detected cases excluded, figure 4D).

**Table 4:** Trends in colorectal cancer stage 1975–2012.

	All cases					Non-neoadjuvant cases				Colon cancer				Colon cancer excluding screen detected cases			
	Dukes A	Dukes B	Dukes C	na^1^	nd^2^	Dukes A	Dukes B	Dukes C	na^1^	Dukes A	Dukes B	Dukes C	na^1^	Dukes A	Dukes B	Dukes C	na^1^
1975 (n = 119)	10.1%	40.3%	31.9%	17.6%		10.1%	40.3%	31.9%	17.6%	9%	39.7%	28.2%	23.1%	9%	39.7%	28.2%	23.15
1980 (n = 179)	6.1%	50.3%	36.9%	6.7%		6.1%	50.3%	36.9%	6.7%	3.9%	53.9%	35.2%	7%	3.9%	53.9%	35.2%	7%
1985 (n = 163)	11%	41.7%	42.9%	4.3%		11%	41.7%	42.9%	4.3%	7%	51.3%	37.4%	4.3%	7%	51.3%	37.4%	4.3%
1990 (n = 188)	8%	50.5%	39.4%	2.1%		8%	50.5%	39.4%	2.1%	5.9%	55.1%	38.2%	0.7%	5.9%	55.1%	38.2%	0.7%
1995 (n = 173)	8.7%	47.4%	39.4%	4.6%		8.7%	47.4%	39.4%	4.6%	6.4%	46.3%	41.8%	5.7%	6.4%	46.3%	41.8%	5.7%
2000 (n = 216)	13%	46.3%	38.9%	1.9%		13%	46.3%	38.9%	1.9%	13.4%	49.2%	38.4%	1.8%	13.4%	49.2%	38.4%	1.8%
2005 (n = 245)	12.2%	42%	42%	-	3.7%	9.9%	45.8%	44.3%	-	8.2%	42.9%	48.9%	-	7.1%	42.4%	50.6%	-
2006 (n = 244)	15.2%	37.7%	43.9%	-	3.3%	9.6%	40.6%	49.7%	-	9.7%	39.8%	50.5%	-	8.9%	40.8%	50.3%	-
2007 (n = 262)	14.1%	38.2%	43.5%	-	4.2%	8.4%	41.4%	50.2%	-	8.1%	41.1%	50.8%	-	7.8%	41.7%	50.5%	-
2008 (n = 298)	22.5%	34.2%	38.3%	-	5%	18.2%	37.7%	44.1%	-	13.9%	39.8%	46.3%	-	14.8%	39.1%	46.2%	-
2009 (n = 278)	16.9%	40.6%	38.1%	-	4.3%	15.1%	43.6%	41.3%	-	13.5%	44.8%	41.7%	-	11.8%	44.4%	43.8%	-
2010 (n = 270)	15.6%	43.7%	36.3	-	4.4%	15.15	48.3%	36.6%	-	14%	48.7%	37.3%	-	9%	49%	41.9%	-
2011 (n = 284)	12.3%	38%	45.1%	-	4.6%	11.6%	39.2%	49.1%	-	9.8%	39%	51.2%	-	8.1%	40.75	51.25	-
2012 (n = 297)	14.8%	39.4%	42.1%	-	3.7%	12.2%	41.5%	46.3%	-	10.5%	42.95	46.6%	-	6%	45.2%	48.%8	-

1. No lymph nodes assessed or no comment about lymph node positivity.

2. Complete pathologic response (i.e. ypT0N0) following neoadjuvant therapy.

**Figure 5:**
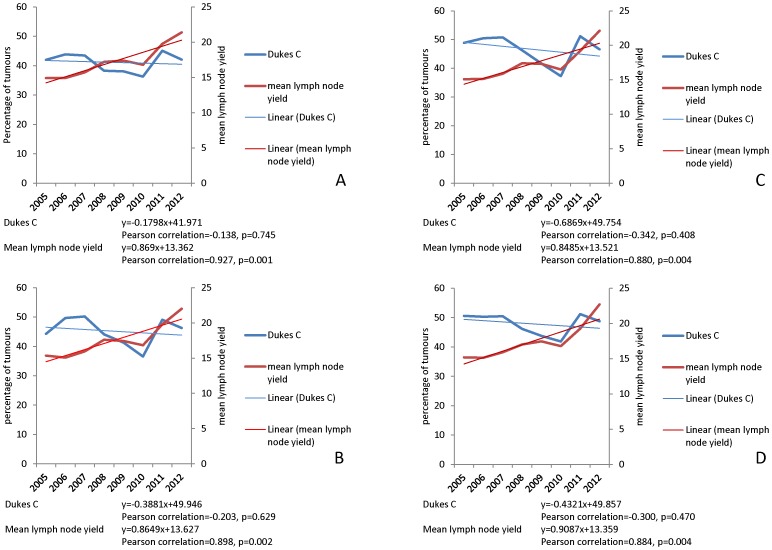
Trends in frequency of Dukes C colorectal cancers and mean lymph node yield 2005–2012. A. All colorectal cancers, B, cases that did not receive neoadjuvant therapy, C. colon cancer, D. colon cancer excluding screen detected cases. For all groups there is a highly significant trend to increasing lymph node yield over this time period without a corresponding trend to an increased proportion of Dukes C/lymph node positive cases. The equations for the linear trend lines for Dukes C cancer and lymph node yield respectively are shown below the corresponding graph.

In the contemporary data set comparison of mean lymph node yield and proportion of Dukes C cases shows a highly significant year on year increase (Pearson correlation = 0.927, p = 0.001) in lymph node yield in contrast there is no significant trend in Dukes cases (Pearson correlation = −0.138, p = 0.745). This is true both for all colorectal cancers and also when potential confounding factors are excluded from the analysis including administration of neoadjuvant therapy, bowel cancer screening detected cases (Figure 5). Figure 5d shows the trends in lymph node yield in Dukes C cases and mean lymph node yield in colon cancer with those confounding factors excluded from the analysis there remains a significant upward trend in mean lymph node yield (Pearson correlation = 0.884, p = 0.004) while there is a non-significant trend towards a lower proportion of Dukes C cases (Pearson correlation = −0.300, p = 0.470).

## Discussion

This large study from a single centre assesses the trend in lymph node evaluation over a thirty seven year period. It utilises both a contemporary dataset and a historic dataset to compare the effect of time and recent advances in colorectal cancer treatment, screening and pathological reporting on lymph node yield and involvement. It examines in detail the effect lymph node yield has on the detection of lymph node positive disease and hence colorectal cancer staging. All the data has been compiled from one centre thus minimising potential data collection and reporting bias.

Factors that influence lymph node yield include patient, surgeon oncologist and pathologist and in designing this study it was important to consider key confounding factors to allow meaningful comparison of data especially between the historic dataset and the contemporary dataset. [10]–[12] The major changes in colorectal cancer diagnosis and management that have occurred and have been considered are the introduction of bowel cancer screening and the use of neoadjuvant therapy as standard therapy for rectal cancer judged at high risk of local recurrence. NHS Grampian was a pilot site for assessment of the bowel cancer screening programme from 2000–2006 prior to the Scottish wide introduction of this programme in 2007. The introduction and widespread adoption of neoadjuvant therapy as standard therapy for rectal cancers judged at high risk of local recurrence (using thin slice MRI) was the standard of care in this region by 2005. Bowel cancer screening has the potential to alter the stage distribution of colorectal cancer with the detection of more early stage tumours while neoadjuvant therapy for rectal cancer would be expected to alter stage distribution and also potentially reduce lymph node yield. [13]–[18] Information regarding both these factors was available for patients in the contemporary dataset and both these factors have been explicitly considered in the analysis of the data. There is also the expectation that in a well screened population over time there should be a reduction in the number of lymph node positive colorectal cancers. This explanation for the changes in the frequency of Dukes C cancer is thought to be unlikely as the bowel screening programme has probably not been implemented for a long enough time period nor during that period has, unfortunately, there been sufficient population participation in the programme. [19]

Histopathological reporting of colorectal cancers has greatly improved over the last 15 years driven by the introduction of guidelines and datasets for the reporting of colorectal cancer excision specimens and the parallel requirement for high quality pathology reporting as adjuvant and neoadjuvant therapy became recognized and established therapeutic options in the treatment of colorectal cancer. [1], [10] It is also important to have well characterised colorectal cancer datasets for translational research e.g. colorectal cancer biomarker research. [20]–[22]

One of the key quality parameter in colorectal cancer histopathology is lymph node yield. The current Royal College of Pathologists UK colorectal cancer dataset recommends a mean of 12 lymph nodes and Healthcare Improvement Scotland quality performance indicator for colorectal cancer pathology is that greater than 80% of colorectal cancers should have 12 or more lymph nodes identified. [10], [11] However, in both guidelines it is either explicitly stated or implied that it is essential to identity all lymph nodes. The rationale for this guidance is that it is assumed that increasing the yield of lymph nodes will increase the proportion of lymph node positive cases being identified. However studies have suggested that this may not be the case and also the identification of what could be described as low volume metastatic disease has led to the development of lymph node ratio as a concept and therapeutic decision making criterion (currently subject of separate ongoing study in this patient population). [23]–[30]

Our data from the historic database shows that from 1975 to 2000 there is a higher proportion of Dukes B tumours and a lower proportion of Dukes C tumours compared to the tumours excised between 2005 and 2012 and this reflects the low lymph node yield per case and supports the concept of understaging of colorectal cancers when there is a low yield of lymph nodes. However in the current data set while there is a significant trend to increase lymph node yield this has not been reflected in an increasing proportion of Dukes C cases even when the confounding factors of neoadjuvant therapy and bowel cancer screen detected cases have been excluded from the analysis. The reasons for this are not apparent and have been observed in other series. Age distribution and anatomical site distribution of tumours within the colon both of which are other potential confounding factors do not appear to have significantly altered with time. Over the period of time from 2005–2012 pre-operative staging protocols have also remained constant. This may suggest that there may be a threshold for lymph node yield above which there is marginal benefit in identifying further lymph nodes. [31] Nonetheless at the practical level there is still strong justification for ensuring all lymph nodes are identified in a colorectal cancer excision specimen. Lymph node yield is a valuable quality parameter for both pathology and surgery and a low lymph node yield appears to be associated with poorer prognosis. [32] Any use of lymph node ratio as a prognostic factor is also predicated on optimal identification of both all involved lymph nodes and the total number of lymph nodes. However, it also suggests that other prognostic markers need to be considered and given more significance in the decision making process of colorectal cancer treatment including the design of future clinical trials. These include other known prognostic parameters especially extramural venous invasion, further studies of ratio of involved to total lymph nodes and the selective use of validated prognostic biomarkers. [33]
